# Multifunctional Hydrogel Nanocomposites for Biomedical Applications

**DOI:** 10.3390/polym13060856

**Published:** 2021-03-11

**Authors:** Emma Barrett-Catton, Murial L. Ross, Prashanth Asuri

**Affiliations:** Department of Bioengineering, Santa Clara University, Santa Clara, CA 95053, USA; ebarrettcatton@scu.edu (E.B.-C.); mross@scu.edu (M.L.R.)

**Keywords:** multifunctional, hydrogel nanocomposties, tissue engineering, drug delivery, wound healing, bioprinting, biowearable devices

## Abstract

Hydrogels are used for various biomedical applications due to their biocompatibility, capacity to mimic the extracellular matrix, and ability to encapsulate and deliver cells and therapeutics. However, traditional hydrogels have a few shortcomings, especially regarding their physical properties, thereby limiting their broad applicability. Recently, researchers have investigated the incorporation of nanoparticles (NPs) into hydrogels to improve and add to the physical and biochemical properties of hydrogels. This brief review focuses on papers that describe the use of nanoparticles to improve more than one property of hydrogels. Such multifunctional hydrogel nanocomposites have enhanced potential for various applications including tissue engineering, drug delivery, wound healing, bioprinting, and biowearable devices.

## 1. Introduction

Hydrogels have broad applicability in the biomedical industry due to their tunable physical and biochemical properties (Table 1) [1,2,3]; however, their poor mechanical strength limits their uses to non-load bearing applications [4,5,6,7,8,9]. Therefore, the development of strategies to improve mechanical properties as well as add or improve other complementary properties has been a focus of research for the past few decades. Researchers have investigated improving the mechanical properties of hydrogels via multiple approaches including, but not limited to, the incorporation of nanoparticles or the addition of a second polymer network to form interpenetrating- or double-network (IPN or DN) hydrogels [2,10,11,12,13,14,15,16]. Nanoparticles (NP) are thought to improve the mechanical properties of hydrogels by forming links with the polymer chain that can desorb under stress, relieving some of the tension within the hydrogel network [10,17,18]. Researchers have also performed experiments in support of the hypothesis that nanoparticles can contribute to the overall degree of crosslinking within the polymer network and thereby lead to enhancements in their mechanical properties [15,16,19,20,21,22,23]. Improvements in the mechanical properties of hydrogels have been shown using an assortment of nanomaterials including clay [17,18,21,24], carbon-based [19,23], polymeric [25,26,27,28], metal [6], and metal oxide [29] NPs (Table 2). Studies have also demonstrated enhancements in mechanical properties for IPN hydrogels, which are combinations of two different crosslinked polymers where at least one of the polymers is synthesized or crosslinked in the presence of the other polymer [4,22,30]. DN hydrogels are a specific subset of IPN hydrogels, whose excellent mechanical performances originate from the combination of two polymer networks with contrasting structures, one with a network of brittle sacrificial crosslinks, and another composed of flexible, loosely crosslinks [20,31,32].

**Table 1:** Representative examples of hydrogels used in the biomedical industry and their properties.

Hydrogels	Relevant Properties
Agarose	bioinert [33,34], biocompatible [34], compatible with extrusion-based bioprinting [33], resistant to bacterial adhesion [34]
Alginate	adhesive [35], biocompatible, biodegradable, in vivo stability [36]
Chitosan	antibacterial [26], biodegradable [26,37,38], biocompatible [37], in situ gelation, in vivo immunostimulatory potential [39]
Gelatin Methacrylate	biocompatible, biodegradable, low hemostatic potential, nonimmunogenic [40]
Polyethylene glycol	biocompatible [29], low immunogenicity, low-protein binding [41]
Poly(N-isopropylacrylamide)	adhesive, optically transparent, thermoresponsive [21,42]
Polyvinyl alcohol	biocompatible [43], chemically stable [44], low friction coefficient [43]

**Table 2:** Representative examples of nanomaterials described in this review and their effects on hydrogel properties.

Nanoparticles	Examples of Hydrogel Properties Affected by Nanoparticles
Carbon-based nanomaterials	electrical conductivity [45,46,47], photothermal behavior [45,47], resistance to degradation [48]
Cellulose-based nanomaterials	injectability/printability [37,49], mechanical strength [50], nutrient permeability, self-healing [37]
Graphene Oxide NPs	injectability/printability [43], stimuli-responsive [51], drug loading [52]
Hydroxyapatite NPs	biomineralization, osteoconductivity/osteoinductivity, self-healing [53]
Silica NPs	mechanical stability, printability [33,51], thermal stability [41], drug loading [54,55]
Silver NPs	antimicrobial [39,44,52,56,57,58,59], antiviral [60], electrical conductivity [61]

**Figure 1:**
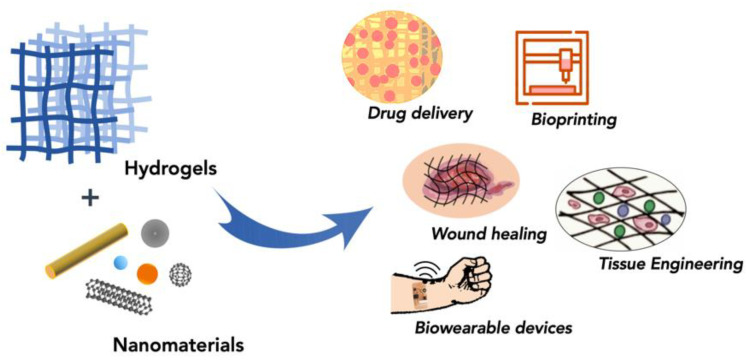
Schematic showing the broad applicability of hydrogel nanocomposites in the biomedical industry.

The chemical properties of hydrogels such as sensitivity to stimuli and bioactivity are an integral aspect of their applicability for biomedical use [6,21,33,45,62]. Therefore, it is not surprising that several researchers have investigated whether the incorporation of nanoparticles may also improve the chemical properties of hydrogels to expand their utility for other applications (Table 2) (e.g., drug delivery, tissue engineering, adhesives, bioprinting and biowearable devices (Figure 1) [6,17,18,19,21,23,24,25,26,27]). For instance, it has been shown that not only can the incorporation of clay NPs lead to improvements in the mechanical properties of hydrogels in a concentration-dependent manner, but it can also impact a variety of other properties including sensitivity to stimuli and biocompatibility [17,18,21,24]. By improving these properties, the hydrogel-clay nanocomposites have been shown to be optimal for use in applications such as tissue engineering, adhesives, and bioprinting [28,29,34]. Similar observations have been made for polymeric NPs, which have been shown to improve the mechanical strength of hydrogels as well as their sensitivity to stimuli for drug delivery applications [25,26]. Researchers have also explored incorporating nanoparticles to enhance the electrical and thermal properties along with the mechanical properties of hydrogels (Table 2) [19,23,45,46,50]. For instance, carbon-based nanomaterials have been shown to improve the electrical and thermal properties of hydrogels and extend their applicability for the development of wearable devices; carbon nanotubes can be incorporated to add thermal stability and electric conductivity, while graphene oxide can add thermal responsiveness [19,23,45,46,50]. Nanoparticles have also been used to improve the properties of IPN and DN hydrogels for a wide variety of applications (e.g., tissue engineering, antimicrobial hydrogels, and wearable devices) [5,14,22,31,34,41]. Researchers have investigated using clay, oxide, and quantum dot nanoparticles to improve the properties of IPN and DN hydrogels [5,20,22,31,34,41]. Kheirabadi et al. investigated the addition of modified bentonite, a clay NP, to IPNs, and found that the resulting IPN hydrogel had a higher Young’s modulus and a reduced swelling capacity [22]. Olad et al. demonstrated that the incorporation of clinoptilolite, an oxide NP, into a starch-based semi-IPN could develop hydrogel composites with improved mechanical and swelling properties [20].

**Figure 2:**
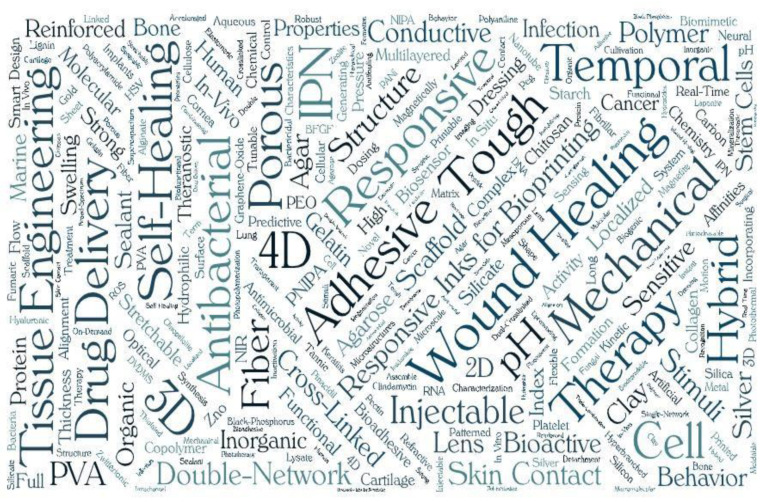
A word cloud generated using the titles of the articles referenced in the review to highlight the broad applicability of multifunctional hydrogel nanocomposites in biomedical sciences and engineering. Word size approximately scales with the frequency of occurrence to highlight the key application areas.

A common theme among the aforementioned examples and the focus of this review is the improvements in more than one property of hydrogels due to the incorporation of nanoparticles, allowing their use for biomedical applications and differentiating our work from others. By improving and adding to the physical and biochemical properties beyond simply the mechanical properties, hydrogel nanocomposites have found broad applicability in biomedical sciences and engineering including use as adhesives, drug delivery agents, antimicrobial dressings, responsive inks for bioprinting, implantable scaffolds, flexible conductive sensors, and cell culture systems, as highlighted in Figure 2 [5,26,46,47,49]. It is important to note that other materials including aerogels, porous gel scaffolds filled with gas (instead of water), have broad applicability in biomedical contexts similar to nanocomposite hydrogels, but are outside the scope of this review and have been discussed by others [63,64,65].

## 2. Tissue Engineering

Hydrogels are ideal for tissue engineering applications given their highly aqueous nature, porous structure, low toxicity, and ability to mimic human physiology [5,32,37,42,54,66]. However, their applicability may be limited due to different factors, including poor mechanical [5,28,32,42,54,67] and optical properties [42]. The addition of nanoparticles addresses these shortcomings of hydrogels, as well as enables the addition of adhesion sites [32,41,42,54] and the delivery of growth factors to support cell growth [54,66]. Furthermore, nanoparticles can add other beneficial properties to hydrogels, such as thermal stability [37,41], self-healing properties [37,67], and promotion of stem cell differentiation [5,37,41], and thereby enable applications in the development of in vitro cell culture platforms and implantable tissue scaffolds.

### 2.1. Cell Culture

While several research groups have investigated the use of various hydrogel nanocomposites for cell culture applications [28,42,54], one hydrogel, poly(N-isopropylacrylamide) (PNIPA), has received particular attention for cell culture [28,42]. PNIPA is particularly suited for tissue engineering because it is thermosensitive, which allows researchers to detach cell sheets without the use of trypsin, thereby keeping the cell sheets intact [28,42]. Additionally, researchers have demonstrated the ability to create microchannels with controllable flow systems within PNIPA hydrogels, further extending their applicability in tissue culture [28,68]. However, similar to most other hydrogels, PNIPA has poor mechanical, adhesive, optical, and swelling properties, which limits their broad applicability [28,42]. Haraguchi et al. added hectorite, a type of clay NP, to PNIPA hydrogels and found that the nanoparticles improved the hydrogels in all of the aforementioned areas, thus improving their suitability for cell culture and tissue engineering applications [28,42]. Nanoparticles may also be added to other types of hydrogels such as gelatin methacryloyl (GelMA) to enable their applicability for cell culture applications including 3D cell culture [54]. For example, researchers incorporated silica NPs into GelMA to control its mechanical properties and enable it to guide differentiation of stem cells derived from bone marrow. Furthermore, the nanoparticles aided with loading and sustained release of pinacidil, a type of vasodilator that aids in cell adhesion, to improve cell viability [54].

### 2.2. Implantable Tissue Scaffolds

The use of hydrogel nanocomposites has also been investigated for the development of implantable tissue scaffolds, either via injection or surgical insertion [32,37,53,55,66,67,69]. For instance, Hu et al. created an injectable tissue scaffold by incorporating pH-sensitive acetylated β-cyclodextrin (Ac-β-CD) NPs loaded with the growth factor VEGF165 into hyaluronic acid (HA)-furan hydrogel, which improved cell growth and viability in the hydrogel. Additionally, the gelation time for the nanocomposite gels was pH dependent, which lends to the development of facilely injectable hydrogels [66]. Basu et al. developed an implantable DNA hydrogel with silicate nanodisks that released the chemokine, SDF-1α, over an extended period of time to recruit stem cells to the wound site and improve healing time [55]. Other researchers have explored the use of hydrogel nanocomposites as implantable scaffolds for cancer treatment [69], artificial bone grafts [53], and neural regeneration [37]. Wang et al. designed injectable polyvinyl alcohol (PVA) hydrogels for gene therapy by complexing nanoparticles with DNA to trigger the production of apoptotic proteins in tumor cells [69]. Hydrogel scaffolds can also be incorporated with hydroxyapatite NPs and recombinant proteins to add osteoconductivity and self-healing properties to develop artificial bone grafts that can encapsulate adipose-derived stem cells for immediate implantation [53]. Cheng et al. incorporated cellulose nanofibers (CNF) into chitosan hydrogels for neural regeneration applications and found that the CNF decreased the self-healing time, prolonged the degradation time, and improved neural stem cell viability and differentiation [37]. They implanted the hydrogel in cerebellar injured zebrafish and found that the chitosan-CNF hydrogel increased fish survival and recovery rates compared to the control group [37], thereby demonstrating that nanoparticle mediated delivery of chemicals and biologicals to control cell fates enhances the applicability of hydrogels for injectable tissue scaffolds.

### 2.3. Interpenetrating Polymer Network (IPN) or Double-Network (DN) Tissue Engineering Applications

The addition of nanoparticles to hydrogels for tissue engineering applications can also be extended to interpenetrating polymer network (IPN) and double network (DN) hydrogels [5,41]. Patel et al. created a cell culture microarray that can be used to study cell behavior and survival using maleimide functionalized polyethylene glycol (PEG-MAL)—gelatin IPN that was ionically cross-linked with silicate NPs. The silicate NPs improved the mechanical and thermal stability of the hydrogel and enhanced its ability to promote cell adhesion and induce osteogenic differentiation [41]. Other researchers used various DN hydrogels in conjunction with black phosphorus nanosheets to facilitate bone regeneration [5]. DN hydrogels are already well suited for bone tissue applications because the DN improves the mechanical strength of the hydrogel, rendering it more comparable to bone extracellular matrix (ECM) [5]. However, in addition to further improving the mechanical strength of the hydrogel (beyond the effect of the DN), the incorporation of black phosphorus promoted stem cell differentiation into osteoblasts and facilitated CaP crystal formation on the hydrogel [5]. CaP crystals are an important part of the bone ECM, so by aiding CaP crystal formation, the nanoparticles enabled the hydrogel to better mimic the natural bone environment and aid with bone regeneration [5].

## 3. Drug Delivery

On-demand delivery is a form of drug delivery where a stimulus, either generated in the body or administered externally, causes drug release [70]. Hydrogels can be tailored to respond to stimuli, and therefore are optimally suited for the localized delivery of drugs to more effectively target tumor cells or various pathogens [26,38]. However, without manipulation, many hydrogels may release drugs in an uncontrolled or unpredictable manner [25,26]. The addition of nanoparticles can provide control over the degree of crosslinking and/or porosity, thereby improving their application in drug delivery and injectability. For instance, a nanocomposite hydrogel was developed for long-term drug delivery of biomacromolecular drugs in the inner ear by incorporating a protein drug into poly(lactic-co-glycolic acid) (PLGA) NPs, which improved mechanical properties of the hydrogel as well as extended the time of drug release 1.5- to 3-fold [48]. Nanoparticles may also enable the development of hydrogels to respond to a variety of in situ and external stimuli including electromagnetic radiation, thus allowing the simultaneous use of orthogonal delivery modalities [25,26,38,70].

### 3.1. In Situ Stimuli

One common in situ stimuli for controlled drug release is pH, as tumors and infected tissues often have a different pH to the healthy surrounding tissue [25,26,70]. Wei et al. designed a chitosan nanocomposite using hyperbranched NPs loaded with the antibiotic clindamycin, which allowed for the controlled delivery of the antibiotic in high pH environments for extended periods of time [26]. The prolonged antibiotic treatment killed ~90% of bacteria present including *E. coli*, *Staphylococcus aureus*, and Methicillin-resistant *S. aureus* (MRSA), without the risk of forming antibiotic-resistant bacteria [26]. In a different approach, Dai et al. combined silver NPs (AgNPs) with cationic dendrimers, both of which are antimicrobial substances, in a pH-responsive hydrogel to enhance antimicrobial efficacy against *E. coli*, *Pseudomonas aeruginosa*, *Staphylococcus epidermidis*, and *S. aureus* in acidic environments [39]. Researchers have also designed pH responsive hydrogel nanocomposites to treat solid tumors. The delivery properties of the hydrogel could be fine-tuned using different nanoparticle–drug ratios to enable independent control over the delivery of multiple drugs at the tumor site, without leading to systemic toxicity as caused by traditional intravenous chemotherapy [25]. When the hydrogel was injected into mice xenografted with A549 lung cancer cells, tumor growth was significantly inhibited when compared to an injection of the same dose of drugs [25], showcasing the ability of nanocomposite hydrogels to reduce the reliance on the systemic delivery of drugs. Hydrogel nanocomposites that respond to changes in pH can also be utilized for the oral delivery of protein-based drugs. For instance, Mamidi et al. designed a zein protein hydrogel with carbon nano-onions that can resist degradation under low pH conditions found in the stomach and upper intestinal tract and deliver proteins to the neutral environment of the colon [71].

### 3.2. External Stimuli

Other researchers have developed on-demand delivery systems that respond to external stimuli such as electromagnetic radiation, which allows for more control over the timing and amount of drug released for localized and recurring dosage for the treatment of infections, post-surgery wounds, and cancers [38,70]. Ultraviolet (UV) radiation is often required to cause structural changes in hydrogels to deliver drugs; however, unlike near-infrared (NIR) radiation, UV cannot penetrate deep into the tissue and is carcinogenic [70]. NIR does not deliver adequate energy, and thereby cannot be used directly to dissolve hydrogels for drug delivery [70]. However, with the addition of nanoparticles, like lanthanide-doped LiYF4 NPs, hydrogels can locally upconvert NIR to UV wavelengths, enabling drug delivery [70]. Others have leveraged the photothermal effects of nanoparticles in combination with chemical drug delivery to create “localized combinatorial therapy,” which is especially helpful in fighting cancer [38]. For example, Xia et al. developed chitosan hydrogels containing silicon-gold NPs that exhibited photothermal effects under NIR for hyperthermia ablation of tumors and aided with the controlled release of anticancer drugs in the acidic environment of the tumor [38]. RNA-based hydrogel nanocomposites have also been shown to treat cancer; nucleic acid NPs and photosensitizers may be incorporated into RNA hydrogels to suppress tumor angiogenesis, promote cell apoptosis, and generate reactive oxygen species (ROS) under light irradiation to increase susceptibility of tumor cells to chemotherapeutic drugs [72].

## 4. Wound Healing

Traditional dressings and sealants often require the use of antibiotics to prevent microbial infection [40]. Researchers have demonstrated that the incorporation of nanoparticles can endow hydrogels with antipathogenic properties [34,39,44,56,73], as well as add angiogenic, or adhesive properties [27,29,35,40,74,75], thus improving their use for wound healing [57,76,77].

### 4.1. Antimicrobial Dressings

Some metal and metal oxide NPs are naturally bioactive and can add antipathogenic properties to hydrogels as well as improve mechanical and chemical properties [34,39,44,56,58,60,73]. AgNPs are commonly used for the development of antibacterial, antiviral, and antifungal hydrogels due to the harsh attack of AgNPs on the microbial respiratory chain that prevents cell division, and the inability of microorganisms to develop resistance to silver [57,61,67,71]. For example, chitosan-PVA, carrageenan, and DNA hydrogels modified with AgNPs showed a significant reduction in a variety of bacteria including *Escherichia coli* and *Bacillus* [44,56,59]. Hydrogels with AgNPs can also be used to treat viral infections; for instance, a tannic acid (TA) modified AgNP mucoadhesive hydrogel was used to significantly reduce herpes simplex virus type 1 and 2 infectability after incubation for 24 h [60]. The presence of TA-AgNPs impacts viral attachment and impedes penetration and cell-to-cell transmission [60]. Similar to AgNPs, metal oxide NPs such as zinc oxide (ZnO) or iron oxide impart antimicrobial properties to hydrogels [34,73]. Not surprisingly, the incorporation of ZnO and Ag NPs as well as hyperbranched polyethyleneimine (HPEI) NPs have been shown to prevent the growth of a wide variety of Gram-positive and Gram-negative bacterial strains and fungus, enabling the development of antifouling surfaces [39,78,79].

Researchers are exploring other methods to create antimicrobial wound dressings using nanoparticles including pH sensitive drug delivery [47,76] and the use of phototherapy to generate reactive oxygen species (ROS) [36,57]. Zhu et al. incorporated lignin-based NPs loaded with trans-resveratrol, a drug with antioxidant, immunomodulatory, anticancer, and anti-inflammatory activity, to carboxymethyl chitosan hydrogels. The addition of these nanoparticles not only improved mechanical properties to create a sprayable hydrogel, but also released trans-resveratrol when exposed to a pH stimulus [76]. Another research group incorporated carbon nanotubes (CNTs) and antibiotic moxifloxacin hydrochloride into chitosan hydrogels to deliver the antibiotic on-demand with pH changes in the environment [47]. The CNTs also added photothermal effects to kill bacteria and electrical conductivity to improve cell proliferation, differentiation, regeneration, and accelerate healing [47]. Other research groups have utilized phototherapy to treat wounds by incorporating zeolite imidazolate framework-8 NPs or porphyrin photosensitizer sinoporphyrin sodium (DVDMS) NPs, in addition to adding other biologics or growth factors to improve wound healing [36,57]. In phototherapy, the nanoparticles produce ROS that cause protein dysfunction and DNA degradation, preventing the formation of drug-resistant bacteria and killing any bacteria present in the wound [39,57,58]. Taken together, nanoparticles may be used either directly to kill microbes or through the delivery of antimicrobial chemicals or ROS, thus providing multiple opportunities to optimize the development of hydrogels for treating wounds.

### 4.2. Adhesive Surgical Sealants and Wound Dressings

Traditional surgical sealants and dressings can lead to microbial infections, cause body fluid leakage, and result in complications that can increase the length of hospitalization [40]. Additionally, traditional sealants are not ideal for sensitive applications, such as ocular, neural, and vascular operations [40]. Hydrogels, on the other hand, can be easily modified to present antimicrobial properties, as outlined previously [34,39,44,56,73], and are also able to absorb fluids, thus minimizing chances of leakage [29,40,75]. Additionally, they mimic human tissues [29] and promote cell proliferation and differentiation [40], making good candidates for surgical sealants. Similarly, incorporation of nanoparticles may enable hydrogels to remain adhered to the skin even in the presence of sweat, which along with the ability of hydrogels to absorb excess moisture, allows for the development of dermatological patches for cataplasm or wound dressing [27]. However, many hydrogels have poor adhesive properties [40,75,80] and may benefit from the incorporation of nanoparticles to improve their adhesive and mechanical properties [27,29,35,40,74,75], and add other related beneficial properties, such as reduced gelation time [29], modified biodegradability [40], and shortened blood clotting time [40]. Liu et al. developed a surgical sealant using a PEG hydrogel endcapped with dopamine mimics that incorporated laponite NPs to further improve the adhesive properties of the hydrogel and reduce its gelation time [29,81]. Rajabi et al. also developed a hydrogel nanocomposite surgical sealant using thiolated gelatin (Gel-SH) and gelatin methacrylate (GelMA) that incorporated polydopamine functionalized laponite (PD-LAP) nanosheets, which not only improved the adhesion, but also provided control over the swelling and biodegradability of the hydrogels [40]. Addition of the nanoparticles helped reduce the blood clotting time through surface-mediated interactions with the proteins and the cells, thus providing an additional property attractive for the development of surgical sealants [40].

## 5. Bioprinting

Hydrogels are well-suited for bioprinting due to their biocompatibility, as well as high viscosity which enables retention of shape fidelity after gelation [33]; however, hydrogels often do not exhibit both properties without significant alteration, limiting their applicability as inks for bioprinting [49]. While synthetic hydrogels can be designed to be highly viscous and better suited for printing, they often lack cell adhesion sites [33,49]. On the other hand, natural polymers have better bioactivity, but poor mechanical stability and flow properties [33,49]. As discussed in previous sections, nanoparticles can serve to both improve the mechanical properties of hydrogels and add cell adhesion sites, which, when combined, can enable improved cell viability post-extrusion and high resolution prints [6,33,43,49,51,62,82,83].

### 5.1. 3D Printing

Nanoparticles have been shown to optimize hydrogel bioinks for 3D printing in various ways: incorporation of laponite nanosilicates into agarose tailored its flow behavior, improved its biocompatibility, and provided sites for cell attachment [33]; cellulose nanocrystals (CNCs) reduced the shear stress on cells encapsulated in platelet lysate hydrogels during the extrusion process, which improved cell viability post-printing [49]; and, the addition of silk fibroin-melanin NPs to PEG-tetraacrylate hydrogels improved cell proliferation and reduced the gel transparency, allowing the use of high resolution 3D-projection stereolithography for 3D printing [82]. Leveraging these improvements, the use of nanocomposite hydrogel inks have been investigated in 3D printing tissues with specific properties [43,51,83]. For instance, the addition of graphene oxide-hydroxyapatite NPs in PVA hydrogels improved its mechanical properties such as viscosity and shear thinning to enhance printability and printing accuracy, ultimately resulting in a hydrogel bioink with appropriate compressive and tribological properties for printing artificial cartilage [43]. Another research group demonstrated the use of graphene nanomaterials for bioprinting with neural stem cells and showed that incorporation of graphene improved neural stem cell viability, increased oxygen consumption rates, and promoted neural differentiation [83]. Other researchers have added NIR-responsive graphene oxide NPs along with laponite nanosilicates (that improved the overall printability of hydrogels) to create stimuli-responsive bioinks for potential drug delivery applications [51].

### 5.2. 4D Printing

Hydrogel nanocomposites can also aid in the development of inks for 4D printing, wherein the printed product continues to develop and mature post-printing [6,62]. For example, Betch et al. embedded iron NPs into an agarose-collagen hydrogel blend to direct the orientation of the collagen fibers using magnets after printing, and thereby develop an in vitro scaffold with alternating layers of aligned and randomly oriented fibers [6]. By mimicking the natural organization of collagen fibers in cartilage, cells cultured in the gels had higher collagen I and II expression compared to cells cultured in scaffolds with either completely aligned or completely random collagen fibers [6]. Another research group used direct ink writing (DIW) to pattern laponite NPs into 2-hydroxyethyl methacrylate (HEMA) hydrogels and direct the spatial attachment of fibroblasts and preosteoblast cells. Incorporation of the nanoparticles also influenced the differentiation of the cells in the 3D scaffold, thus allowing the creation of an attractive platform to spatially pattern cellular development in vitro [62].

## 6. Biowearable Devices

Hydrogels are excellent candidates for the development of biowearable devices, as they are safe [46,84], environmentally friendly [50], flexible, stretchable [46,61], resemble the ECM of human tissue [31], and are easily remolded and reused [46,61]. However, the poor conductivity [33,37,45,46] and optical properties [31] of hydrogels can limit their applicability in biowearable devices. Nanoparticles can improve various properties of hydrogels [27,45,46,61,75] and enable their use not only as individual components (e.g conductive elements or adhesives) [27,45,46,61,75], but also as standalone devices (e.g., contact lenses) [31,52,84].

### 6.1. Biowearables for Ocular Applications

While hydrogels have been widely used in ocular applications, such as corneal implants and contact lenses [1,3], the addition of nanoparticles enable improvements in optical properties as well as the introduction of new modalities such as drug delivery and microbial resistance [31,52,84]. For example, researchers designed a corneal implant using an IPN nanocomposite composed of poly(2-hydroethyl methacrylate) (PHEMA) and poly(acrylic acid) (PAA) IPN [31]. Covalent attachment of Zinc Sulfide NPs to PHEMA polymer network adjusted the refractive index (RI) of the IPN, resulting in a clear hydrogel with an optimal RI that enables the development of corneal implants as a potential alternative to laser-assisted in situ keratomileusis (LASIK) surgery [31]. Other research groups have investigated incorporating nanoparticles into hydrogel contact lenses for use as ocular drug delivery systems [52,84]. For instance, one group demonstrated the use of gelatin NPs in contact lenses for the encapsulation and long-term delivery of a model hydrophilic protein-based drug directly to the eye [84]. A different group incorporated AgNPs and graphene oxide loaded with Vor, an antifungal drug, into a quaternized chitosan hydrogel to treat fungal keratitis [52].

### 6.2. Conductive Components

Multiple research groups have also investigated the use of nanoparticles to add or improve conductive properties in hydrogels, enabling them to relay signals and store energy [27,45,46,61]. For instance, Deng et al. added CNTs to N-isopropyl acrylamide (NIPAM) hydrogels to improve their conductivity and add photothermal behavior, resulting in hydrogels that can monitor human motion due to pressure-dependent conductivity [45]. Other researchers have similarly demonstrated the use of CNTs to improve hydrogel conductivity and capacitance for applications as flexible energy storage devices [46]. Furthermore, other types of nanoparticles, such as gold or silver, can also improve the conductivity of hydrogels [61]. In rare cases, some hydrogels, such as zwitterionic polymer-based hydrogels, may already exhibit good electrical conductivity, but can benefit from nanoparticle-mediated improvements in mechanical properties. Yang et al. incorporated cellulose nanocrystals (CNCs) to improve the mechanical properties of the zwitterionic hydrogel, resulting in a hydrogel that can be used for speech recognition and electrical display applications [50].

## 7. Conclusions

As described here and by others, nanoparticles can improve a wide range of physical properties in hydrogels including mechanical, adhesive, optical, and electrical properties. Furthermore, nanoparticles may be used to enhance the biochemical properties of hydrogels such as biocompatibility and biodegradability as well as to introduce new properties such as microbial resistance and response to stimuli. More importantly, this review underscores that incorporation of nanoparticles can improve or add to more than one property of hydrogels to create multifunctional nanocomposite hydrogels that make them ideal for various applications, as summarized in Table 3. Specifically, based on the findings presented in this review, we believe there is a strong case for the use of hydrogel nanocomposites for tissue regeneration, therapeutic delivery, biowearables, and biofabrication. However, challenges still remain with clinical implementation and commercialization of hydrogels including high production costs, difficulties with scaling from laboratory settings to large production for industry, and batch-to-batch reproducibility, as described by others [1,10,85]. Successful translation of hydrogel-based solutions from lab to market also demands receiving regulatory approvals and establishing standardized approaches for scaled manufacturing, among other requirements [86,87]. Nevertheless, as researchers continue to pursue the use of hydrogel nanocomposites, we expect an increase in their clinical and commercial applications in bioengineering and regenerative medicine.

## Figures and Tables

**Table 3:** Summary of hydrogel nanocomposite applications and nanoparticle-mediated hydrogel improvements.

Field	Applications	Improvements in Properties due to Nanoparticles
Tissue Engineering	Cell Culture	Improved mechanical [28,42,54], adhesive, optical, swelling properties [28,42], directed stem cell differentiation, sustained release of factors [54]
	Implantable Tissue Scaffolds	pH responsive [66], sustained release of factors [55,66,69], guidance of stem cell migration [55], osteoconductive, self-healing [53], decreased healing time [37,69], tailored degradation, improved cell viability and differentiation [37]
	IPN and DN Tissue Engineering	Improved mechanical [5,41] and thermal stability [41], increased cell adhesion [41], directed stem cell differentiation [5,41]
Drug Delivery	In Situ Stimuli	pH responsive [25,26,39,71]
	External Stimuli	Electromagnetic radiation responsive [38,70,72], local upconversion of wavelengths [70], photothermic [38], ROS generation [72]
Wound Healing	Antimicrobial Dressings	Antipathogenic [34,39,44,56,58,60,73], controlled drug delivery [47,76], improved mechanical and chemical properties [34,39,44,56,58,60,73], mucoadhesive [60], decreased pathogen adhesion and infection [34,60], pH responsive [47,76], ROS generation [36,57], electrically conductive [47]
	Adhesive Surgical Sealants	Improved adhesive and mechanical properties [27,29,35,40,74,75], controlled biodegradability and swelling [40], reduced gelation time [29,81], shortened blood clotting time [40]
Bioprinting	3D Printing	Improved optical [82] and mechanical properties for enhanced printability and cell viability [33,43,49,51], increased biocompatibility [33], improved cell attachment [33], proliferation [82,83] and differentiation [83]
	4D Printing	Directed orientation of fibers (within the hydrogel) [6], spatial attachment and differentiation of cells [62]
Biowearable Devices	Ocular Applications	Improved optical properties [31], antipathogenic, localized, long term drug delivery [52,84]
	Conductive Components	Improved electrical [45,46,61], mechanical [50], and photothermic properties [45]

## Data Availability

No new data were created in this study. Data sharing is not applicable to this article.
